# A prognostic classifier for patients with colorectal cancer liver metastasis, based on AURKA, PTGS2 and MMP9

**DOI:** 10.18632/oncotarget.6188

**Published:** 2015-10-20

**Authors:** Jeroen A.C.M. Goos, Veerle M.H. Coupé, Mark A. van de Wiel, Begoña Diosdado, Pien M. Delis-Van Diemen, Annemieke C. Hiemstra, Erienne M.V. de Cuba, Jeroen A.M. Beliën, C. Willemien Menke - van der Houven van Oordt, Albert A. Geldof, Gerrit A. Meijer, Otto S. Hoekstra, Remond J.A. Fijneman

**Affiliations:** ^1^ Department of Pathology, VU University Medical Center, Amsterdam, The Netherlands; ^2^ Department of Radiology and Nuclear Medicine, VU University Medical Center, Amsterdam, The Netherlands; ^3^ Department of Epidemiology and Biostatistics, VU University Medical Center, Amsterdam, The Netherlands; ^4^ Department of Pathology, Netherlands Cancer Institute, Amsterdam, The Netherlands; ^5^ Department of Medical Oncology, VU University Medical Center, Amsterdam, The Netherlands

**Keywords:** colorectal cancer, liver metastasis, prognosis, classifier, biomarker

## Abstract

**Background:**

Prognosis of patients with colorectal cancer liver metastasis (CRCLM) is estimated based on clinicopathological models. Stratifying patients based on tumor biology may have additional value.

**Methods:**

Tissue micro-arrays (TMAs), containing resected CRCLM and corresponding primary tumors from a multi-institutional cohort of 507 patients, were immunohistochemically stained for 18 candidate biomarkers. Cross-validated hazard rate ratios (HRRs) for overall survival (OS) and the proportion of HRRs with opposite effect (*P*(HRR < 1) or *P*(HRR > 1)) were calculated. A classifier was constructed by classification and regression tree (CART) analysis and its prognostic value determined by permutation analysis. Correlations between protein expression in primary tumor-CRCLM pairs were calculated.

**Results:**

Based on their putative prognostic value, EGFR (*P*(HRR < 1) = .02), AURKA (*P*(HRR < 1) = .02), VEGFA (*P*(HRR < 1) = .02), PTGS2 (*P*(HRR < 1) = .01), SLC2A1 (*P*(HRR > 1) < 01), HIF1α (*P*(HRR > 1) = .06), KCNQ1 (*P*(HRR > 1) = .09), CEA (*P* (HRR > 1) = .05) and MMP9 (*P*(HRR < 1) = .07) were included in the CART analysis (*n* = 201). The resulting classifier was based on AURKA, PTGS2 and MMP9 expression and was associated with OS (HRR 2.79, *p* < .001), also after multivariate analysis (HRR 3.57, *p* < .001). The prognostic value of the biomarker-based classifier was superior to the clinicopathological model (*p* = .001). Prognostic value was highest for colon cancer patients (HRR 5.71, *p* < .001) and patients not treated with systemic therapy (HRR 3.48, *p* < .01). Classification based on protein expression in primary tumors could be based on AURKA expression only (HRR 2.59, *p* = .04).

**Conclusion:**

A classifier was generated for patients with CRCLM with improved prognostic value compared to the standard clinicopathological prognostic parameters, which may aid selection of patients who may benefit from adjuvant systemic therapy.

## INTRODUCTION

Annually, 700.000 people diagnosed with colorectal cancer (CRC) die as a consequence of advanced disease [1]. The majority of CRC metastases localize to the liver and resection of the affected liver tissue, often combined with radiofrequency ablation (RFA), is the sole curative treatment option [2]. Selection of patients for liver surgery is usually based on established clinicopathological prognostic variables [3–6]. However, as survival after surgery is only 36–58% [7], the prognostic accuracy of such scoring systems is disputed [8–12]. Accordingly, there is a need for better prognostic variables to select patients for treatment based on their tumor biology [10–12]. Potentially, relevant biological information on colorectal cancer liver metastasis (CRCLM) could be derived from molecular profiling of the corresponding primary tumor, since genomic characteristics of primary and matched metastatic CRC lesions overlap to a large extent [13, 14]. This is of particular interest since specimens of the primary tumor are more often readily available for histopathological analysis.

Proteins involved in key biological processes of CRC have the potency to serve as biomarkers for patient stratification [15]. Such biological processes include sustained proliferation, growth suppressor evasion, apoptosis resistance, stimulation of angiogenesis, invasion and metastasis, genome instability, promotion of inflammation and deregulation of cellular energetics (Table 1) [15]. Genes involved in these processes are often mutated in CRC and/or the corresponding proteins are aberrantly expressed compared to normal tissue [14, 16–30]. Expression of several of such proteins in primary CRC has been associated with survival. However, the prognostic value of most of these proteins has not yet been determined for their expression in CRCLM. We have previously demonstrated that protein expression levels of EGFR, AURKA, VEGFA, PTGS2 and SLC2A1 are independently associated with survival of patients with CRCLM [31–33], and have analyzed the effects of several other proteins commonly associated with CRC (LM). The present study aims to explore how survival of CRCLM patients could be predicted more accurately by combining such markers into a potentially clinically applicable classifier. To this end, we investigated the single and combined prognostic value of proteins commonly associated with CRC carcinogenesis (Table 1) [34–39], Furthermore, we compared biomarker expression between CRCLM and the corresponding primary tumor to assess whether prognosis could be predicted based on expression within the corresponding primary tumor.

**Table 1:** Overview of several biological processes involved in CRC carcinogenesis and proteins commonly associated with these processes

Biomarker	Full name	Sustained proliferation	Growth suppressor evasion	Apoptosis resistance	Angiogenesis	Invasion and metastasis	Genome instability	Inflammation	Deregulation of cellular energetics
EGFR	epidermal growth factor receptor	X	X	X		X			
PI3K	phosphatidylinositide 3-kinase	X	X	X		X			
AURKA	aurora kinase A	X	X	X		X			
Ki-67	antigen KI-67	X		X					
TK1	thymidine kinase 1	X		X					
KCNQ1	potassium voltage-gated channel, KQT-like subfamily, member 1	X	X	X					
IGF2	insulin-like growth factor 2	X	X	X		X			
VEGFA	vascular endothelial growth factor A	X		X	X	X			
PDGFR β	platelet-derived growth factor receptor β	X	X	X	X	X			
CEA	carcinoembryonic antigen		X			X			
MMP9	matrix metallo-peptidase 9	X		X	X	X			
CXCR4	C-X-C chemokine receptor type 4	X		X	X	X			
CXCL12	C-X-C motif chemokine 12	X		X	X	X			
MLH1	MutL homolog 1, colon cancer, nonpolyposis type 2 (E. coli)	X		X			X		
MSH6	mutS homolog 6			X			X		
PTGS2	prostaglandin-endoperoxide synthase 2	X	X	X		X		X	
SLC2A1	solute carrier family 2, facilitated glucose transporter, member 1	X		X		X			X
HIF1α	hypoxia-inducible factor 1α	X	X	X	X	X		X	X

## RESULTS

### Patient characteristics

Patient characteristics of the study cohort have been described previously and are summarized in Supplementary Table S1 [32]. Cumulative five-year overall survival (OS) after CRCLM resection was 41.2% (Supplementary Figure S1).

### Single and combined prognostic value of candidate biomarkers in CRCLM

**Figure 1:**
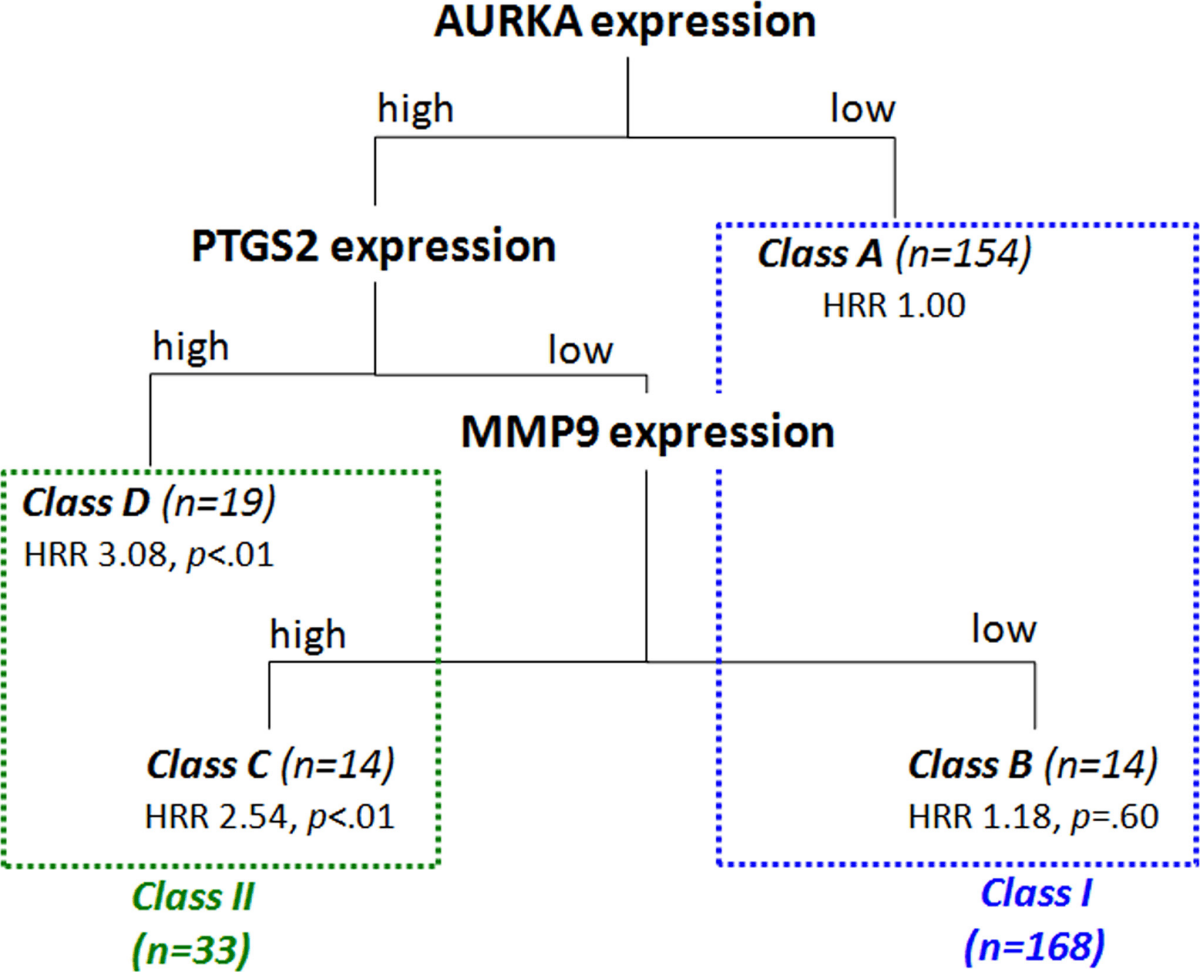
Classification tree resulting from the Classification and Regression Tree (CART) analysis including the nine prognostically most relevant proteins in our study cohort (i.e. EGFR, AURKA, VEGFA, PTGS2, SLC2A1, KCNQ1, CEA, MMP9 and HIF1α) The optimal prediction of three-year survival was obtained by a classification tree including AURKA, PTGS2 and MMP9 expression. Class A contained patients with low AURKA expression, class B patients with high AURKA, low PTGS2 and low MMP9 expression, class C patients with high AURKA, low PTGS2 and high MMP9 expression and class D patients with high AURKA and high PTGS2 expression. Based on the HRRs of the individual classes, classes A and B were grouped and classes C and D were grouped, resulting in classes I and II, respectively. Excluded from analysis were patients of whom no data was available on survival status or three-year survival and with unknown expression of AURKA, PTGS2 and MMP9. HRR: hazard rate ratio, *p*: *p*-value as determined by Cox regression analysis.

**Figure 2:**
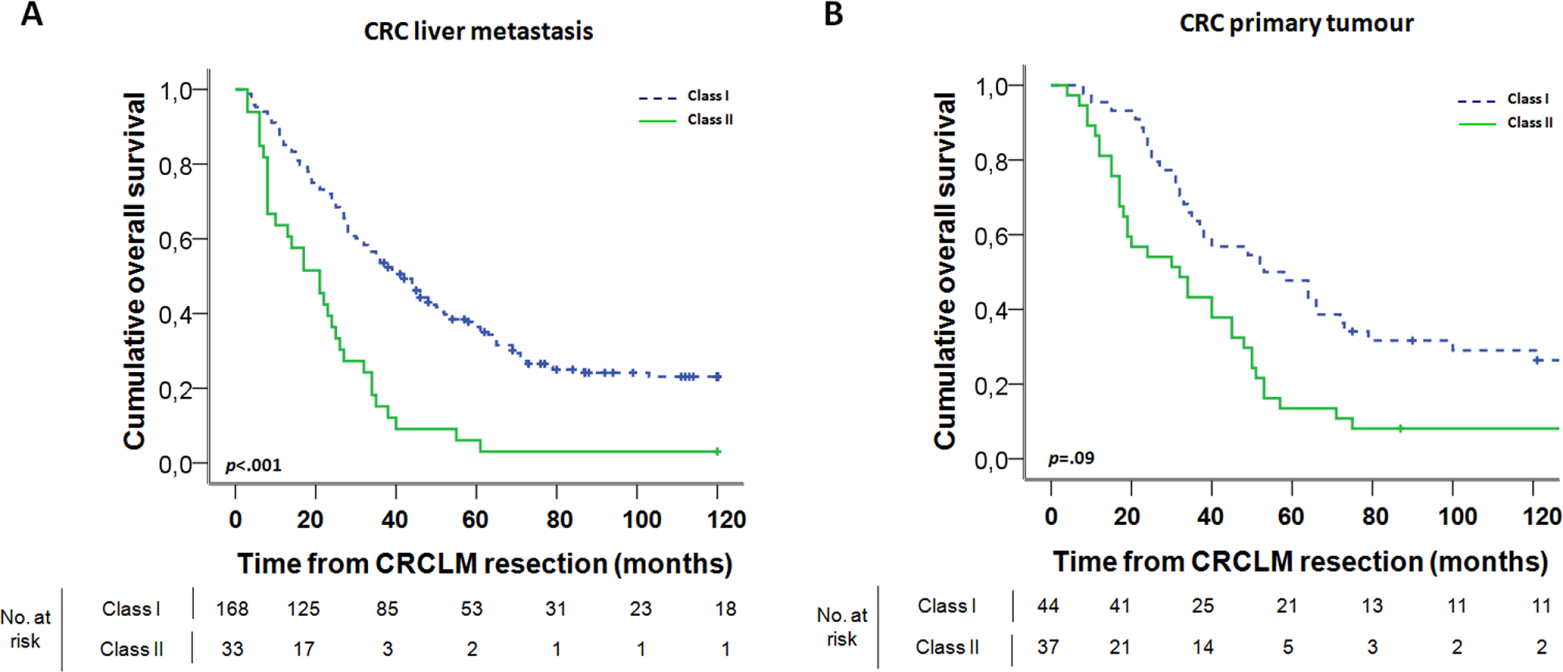
Kaplan-Meier graphs depicting OS in months, stratified by the classes resulting from the CART and Cox regression analyses, based on expression in (A) CRCLM and (B) primary CRC The *p*-value is the corrected *p*-value as determined by permutation analysis. Excluded from analyses were patients with unknown or less than two months survival and with unknown expression of AURKA, PTGS2 and MMP9.

Based on an extensive literature search, eighteen biomarkers were selected which represent key biological processes involved in the development and progression of CRC and have been described as (promising) diagnostic, prognostic, and/or predictive biomarkers. Our selection of investigated proteins included EGFR, PI3K, AURKA, Ki-67, TK1, KCNQ1, IGF2, VEGFA, PDGFR β, CEA, MMP9, CXCR4, CXCL12, MLH1, MSH6, PTGS2, SLC2A1 and HIF1α (Table 1 and Supplementary Figure S2). After correction for the established clinicopathological prognostic variables, high CRCLM expression of EGFR (average hazard rate ratio HRRav 1.54; *P*(crude hazard rate ratio HRR < 1) = .02), AURKA (HRR_av_ 1.66; *P*(HRR < 1) = .02), VEGFA (HRRav 1.50;*P*(HRR < 1) = .02) and PTGS2 (HRR_av_ 1.59; *P*(HRR < 1) = .01) were associated with poor prognosis and high expression of SLC2A1 (HRRav 0.65; *P*(HRR > 1) < .01) was associated with good prognosis, as we reported previously (Table 2 and Supplementary Figure S3) [31–33]. Although the preset threshold for significance was not reached, decreased survival was also suspected for high MMP9 expression (HRRav 1.34; *P*(HRR < 1) = .07) and increased survival for high expression of KCNQ1 (HRRav 0.81; *P*(HRR > 1) = .09), CEA (HRRav 0.63; *P*(HRR > 1) = .05), and HIF1α (HRRav 0.77; *P*(HRR > 1) = .06), the latter as described previously [33]. These nine proteins were selected as prognostically most relevant and were combined in a classification and regression tree (CART) analysis. The lowest misclassification rate for predicting three-year survival (cross-validated error rate 22.5%) was achieved using a classification tree with four classes (Figure 1), based on AURKA, PTGS2 and MMP9 expression in CRCLM (*n* = 201; Supplementary Figure S4A). First, four classes were discerned where class A consisted of patients with low AURKA expression in CRCLM, class B of patients with high AURKA, low PTGS2 and low MMP9 expression, class C of patients with high AURKA, low PTGS2 and high MMP9 expression and class D of patients with high AURKA and high PTGS2 expression. HRRs were calculated for all classes, using class A as reference (Figure 1 and Supplementary Figure S5). Second, classes of patients with similar OS, using class A as reference, were grouped in a single class [40]. Consequently, classes A and B were grouped (HRR < 2.0), and classes C and D were grouped (HRR > 2.0), resulting in class I and class II, respectively. Patient stratification based on these two classes was associated with OS (HRR 2.79, corrected *p* < .001; Figure 2A). Also after multivariate analysis including primary tumor-to-CRCLM interval < 12 months, lymph node positivity at time of diagnosis of the primary tumor, maximal CRCLM diameter > 5.0 cm, number of CRCLM > 1 and serum CEA level > 200 ng/ml as established clinicopathological prognostic variables, classes I and II were associated with OS (HRR 3.57, corrected *p* < .001). As a prognostic model, the classifier based on AURKA, PTGS2 and MMP9 expression was superior to a model only based on the established clinicopathological prognostic variables (*p* = .001).

**Table 2:** Univariate and multivariate average hazard rate ratios of the investigated candidate biomarkers

	univariate			multivariate		
Biomarker	HRR_av_	*P* (HRR < 1)	*P* (HRR > 1)	HRR_av_	*P* (HRR < 1)	*P* (HRR > 1)
EGFR [31]	1.47	.03*		1.54	.02*	
PI3K	0.88		.20	0.80		.20
AURKA [32]	1.57	.02*		1.66	.02*	
Ki-67	1.15	.21		1.17	.20	
TK1	1.22	.26		1.31	.23	
KCNQ1	0.81		.09	0.81		.09
IGF2	0.97		.39	0.96		.37
VEGFA [33]	1.48	.02*		1.50	.02*	
PDGFR β	1.13	.29		1.10	.35	
CEA	0.68		.06	0.63		.05
MMP9	1.29	.08		1.34	.07	
CXCR4	0.94		.33	0.91		.25
CXCL12	0.90		.23	0.93		.31
MLH1	1.16	.23		1.17	.21	
MSH6	0.81		.11	0.82		.13
PTGS2 [31]	1.63	< .01*		1.59	.01*	
SLC2A1 [33]	0.67		< .01*	0.65		< .01*
HIF1α [33]	0.80		.06	0.77		.06

HRR_av_: average hazard rate ratio; *P* (HRR < 1): proportion of HRRs with HRR < 1; *P* (HRR > 1): proportion of HRRs with HRR > 1

* *P* (HRR < 1) or P(HRR > 1) < .05

**Figure 3:**
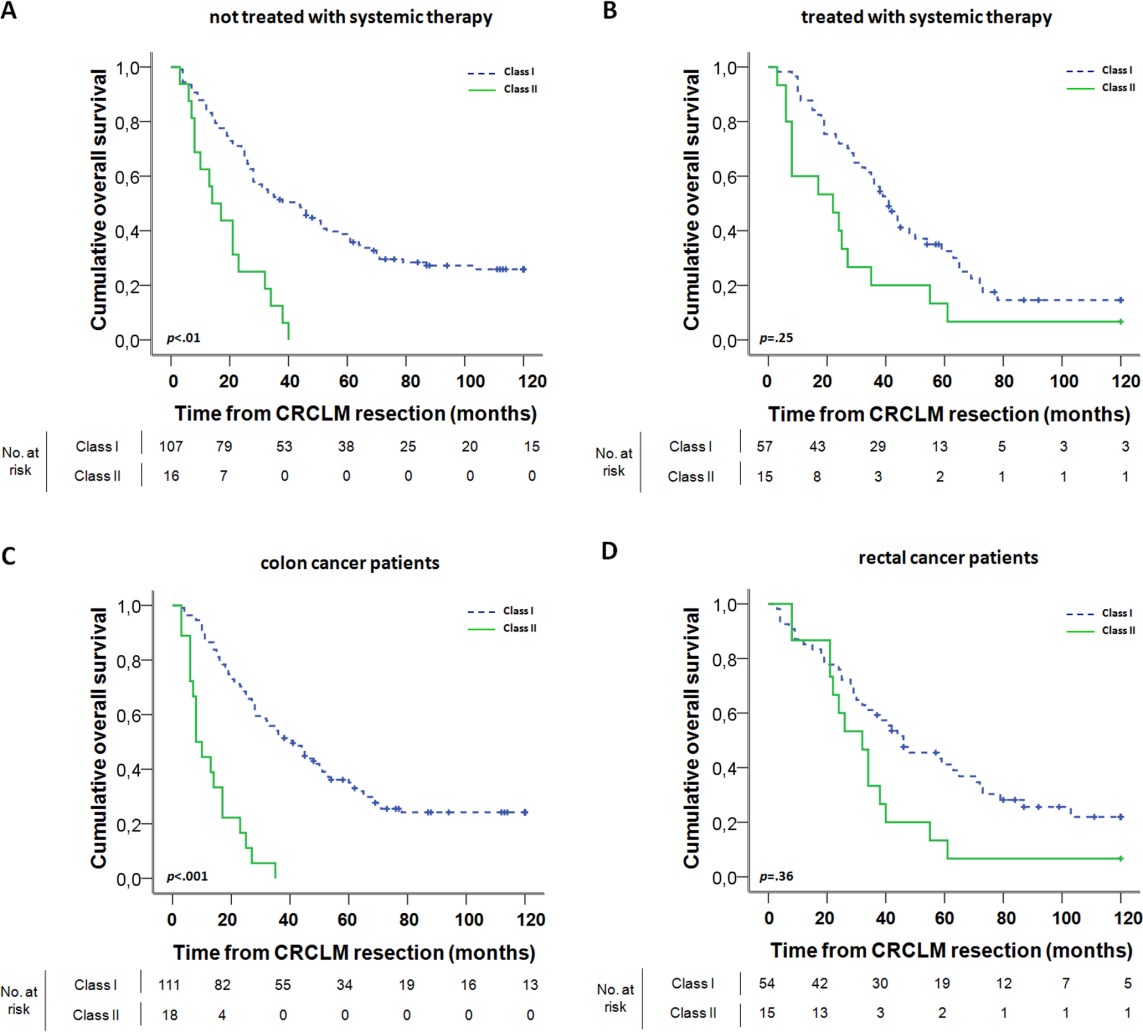
**Kaplan-Meier graphs depicting OS in months of (A).** patients in which liver metastases were not treated with systemic therapy, **(B)** patients in which liver metastases were treated with systemic therapy, **(C)** colon cancer patients, and **(D)** rectal cancer patients, stratified by the classes as identified using the Classification and Regression Trees (CART) analysis. Excluded from analysis were patients with unknown or less than two months survival, unknown systemic therapy or primary tumor localization and with unknown expression of AURKA, PTGS2 and MMP9. The *p*-value is the corrected *p*-value as determined by permutation analysis.

We further evaluated the prognostic value of this classifier after stratifying patients for systemic therapy. In patients not treated with systemic therapy, the classifier was predictive of poor outcome after CRCLM resection (HRR 3.48, corrected *p* < .01; Figure 3A). In patients treated with systemic therapy the classifier lacked prognostic value (HRR 2.16, corrected *p* = .25; Figure 3B). Similarly, we evaluated the prognostic value of the classifier in patients with CRCLM originating from colon cancer and rectal cancer separately. This revealed that particularly in colon cancer patients the classifier was associated with survival (HRR 5.71, corrected *p* < .001; Figure 3C), whereas in rectal cancer patients it was not (HRR 1.95, corrected *p* = .36; Figure 3D).

### Candidate biomarker expression in primary CRC

Primary CRC tissue specimens are often more readily available for pathological examination than CRCLM tissue specimens. Therefore, we compared candidate biomarker expression between primary CRC and corresponding CRCLM and assessed the prognostic value of the classifier in primary CRC. For 12 out of 18 candidate biomarkers (67%), expression in primary CRC and corresponding CRCLM was positively correlated (*r* = 0.20–0.39; Table 3). Next, we investigated whether the classifier could be applied to predict survival according to biomarker expression levels in the primary tumor (*n* = 81; Supplementary Figure S4B). Based on these expression levels, a survival difference was observed between class I and class II patients (HRR 2.11, corrected *p* = .09; Figure 2B), however, the threshold for significance was not reached. MMP9 expression was not correlated between matched primary CRC and CRCLM pairs (Table 3). Omitting MMP9 resulted in a classifier based on AURKA expression only. Classification of patients based on AURKA expression in the primary tumor alone, demonstrated that high AURKA expression in the primary tumor was associated with decreased survival (HRR 2.59, corrected *p* = .04).

**Table 3:** Correlation between candidate biomarker expression in primary CRC and patient-matched CRCLM, as calculated using Pearson's correlation test

Biomarker	*r*	*p*-value
EGFR	0.03	.77
PI3K	0.24	< .01*
AURKA	0.34	< .01*
Ki-67	0.24	< .01*
TK1	0.13	.11
KCNQ1	0.34	< .01*
IGF2	0.27	< .01*
VEGFA	−0.03	.74
PDGFR β	0.31	< .01*
CEA	0.20	.01*
MMP9	−0.04	.68
CXCR4	0.20	.01*
CXCL12	0.39	< .01*
MLH1	0.34	< .01*
MSH6	0.21	.02*
PTGS2	0.20	< .01*
SLC2A1	0.16	.05
HIF1α	−0.03	.68

*r*: Pearson's correlation coefficient

* *p*-value < .05

## DISCUSSION

In a population of CRC patients with resectable liver metastasis, we investigated the prognostic value of 18 proteins, which are commonly associated with CRC(LM) carcinogenesis. We have previously demonstrated that, individually, expression levels of EGFR, AURKA, VEGFA, PTGS2 and SLC2A1 are associated with prognosis in CRCLM patients undergoing surgical resection [31–33]. In addition, using a similar cross-validation procedure, here we indicated that also expression of MMP9, CEA and KCNQ1 showed a survival difference, comparable to HIF1α [33], although for these proteins the threshold for significance was not reached. We combined expression of these nine proteins in a CART analysis and found that the combination of AURKA, PTGS2 and MMP9 expression in CRCLM provided the best prediction of three-year survival after CRCLM resection. Survival was highest for patients with low AURKA expression and for patients with high AURKA expression, but low PTGS2 and low MMP9 expression (class I). Survival was poorest for patients with both high AURKA and high PTGS2 expression, and for patients with high AURKA but low PTGS2 and high MMP9 expression (class II).

The eighteen proteins investigated in this study represent key biological processes involved in CRC (LM) development and progression (Table 1). Additionally, the proteins were selected based on their putative clinical applicability as well as on the availability of adequate immunohistochemical antibodies. Although the selected proteins cover a wide spectrum of biological functions, several others have been described in literature to be involved in CRC (LM). Regardless of which proteins constitute the prognostic classifier, in essence it should outperform the standard clinically applied prognostic parameters. Here, we show that the classifier based on AURKA, PTGS2 and MMP9 expression was retained as independent prognostic parameter after correction for the standard clinicopathological prognostic variables in a multivariate analysis, with improved prognostic value compared to the established clinicopathological prognostic parameters and stronger than when using individual protein biomarkers [31–33]. Furthermore, it outperformed the prognostic model based on the clinicopathological prognostic parameters. As such, clinical assessment of AURKA, PTGS2 and MMP9 expression may stratify CRCLM patients into subgroups with good versus bad prognosis. Based on this stratification, the classifier may aid in selecting patients for adequate treatment after CRCLM resection.

When stratifying CRCLM patients by location of the primary tumor in the large intestine, it turned out that the classifier performed well in patients with liver metastases originating from colon cancer, but not from rectal cancer. This may be explained by the different functional roles of the proteins of the classification system in colon and rectum, e.g. such as has been indicated for MMP9 [41]. In patients treated with systemic therapy, the classifier also lacked prognostic value, which may suggest that commonly applied systemic therapy regimens improve the survival of patients with high expression of AURKA, PTGS2 and MMP9 (Class II). Accordingly, patients with poor prognosis based on this classifier, may benefit from systemic therapy in addition to liver resection. More likely, however, survival of patients in class I is decreased in this subgroup compared to class I patients in the subgroup not treated with systemic therapy. This may reflect a more elaborate metastatic dissemination – often leading to the addition of systemic treatment to surgery alone in order to improve resectability or remove remnant CRCLM – and a high number of CRCLM has frequently been associated with decreased OS. [3–6] It should be noted, however, that these patient subgroups may be too small to draw firm conclusions from.

Remarkably, for the majority of candidate biomarkers (67%) expression was positively correlated between the primary tumor and corresponding CRCLM. These findings are consistent with the common postulate that in CRC molecular alterations acquired during early tumor development are likely to be present also in the metastases [13, 14]. As such, this may indicate that to a certain extent expression in patient-matched CRCLM could be predicted from expression levels in the primary tumor. However, further investigation is required to determine the clinical applicability of such correlations. The classifier could not be applied to predict survival based on expression of AURKA, PTGS2 and MMP9 in the primary tumor. Interestingly, when using AURKA expression only, the survival difference was significant. As such, AURKA expression levels in the primary tumor are indicative of prognosis for patients with resectable CRCLM.

Selecting the nine prognostically most relevant biomarkers for CART analysis may introduce a minor selection bias. However, as only 18 biomarkers were investigated here, this selection effect will be minimal. To avoid overestimation of the prognostic effect of the classifier, we corrected the reported *P* values using a series of permutated datasets. Similar correction of the HRRs requires complex methodology and, therefore, the HRRs reported in this study may be somewhat overestimated. Nevertheless, the survival difference indicated by the classifier is significant, as demonstrated by the corrected *P* values. The optimal combination of biomarkers that compose the final classifier has been assessed via cross-validation, which is an inherent part of the CART analysis. However, to further confirm its prognostic value, external validation of the results is desirable.

In conclusion, we identified and validated a number of proteins with prognostic value based on protein expression in CRCLM and combined these in a classifier which could be used to predict survival after CRCLM resection. Individually, but also combined, these proteins may add value to existing clinicopathological risk scores, by taking into account biological information which is neglected by the standard clinicopathological prognostic variables in current clinical practice. Resection of the CRCLM, possibly in combination with RFA, will remain the preferred treatment option, regardless of the biological profile of the CRCLM. However, such a classifier may aid in selecting patients who may benefit from adjuvant systemic therapy. As such, adding such a biomarker-based classifier to the standard clinicopathological parameters for stratification of patients may contribute to improving survival after CRCLM resection.

## MATERIALS AND METHODS

### Patient study population

Assembly of the study population has been described previously [32]. In brief, clinicopathological information was obtained from in-hospital clinical and pathology databases of 507 consecutive patients with CRCLM who underwent liver resection with curative intent between 1990 and 2010 in one of seven Dutch hospitals affiliated with the DeCoDe PET group. Corresponding formalin-fixed paraffin-embedded (FFPE) tissue samples were collected of one histologically confirmed CRCLM and an adjacent control liver specimen. Additionally, of 234 patients tissue specimens of matching primary tumor and adjacent colon tissue were obtained. Only specimens of patients with histologically confirmed CRCLM were included in the study, while tissue samples of patients with multiple primary tumors were excluded. Collection, storage and use of clinicopathological data and tissue specimens were performed in compliance with the “Code for Proper Secondary Use of Human Tissue in The Netherlands” [42].

### Tissue microarrays

Three core biopsies (diameter 0.6 mm) were taken from morphologically representative areas of each FFPE donor block and transferred into tissue microarray (TMA) recipient paraffin blocks using the 3DHISTECH TMA Master (v1.14, 3DHISTECH Ltd., Budapest, Hungary). A detailed protocol of TMA generation has been described elsewhere [32].

### Immunohistochemistry

Tissue specimens were immunohistochemically stained for proteins involved in the development and progression of CRC (LM), based on the availability of adequate immunohistochemical antibodies and their potential utility for clinical diagnostics or monitoring (Table 1). TMA sections (4 μm) were deparaffinized using xylene and rehydrated with a decreasing alcohol series. Endogenous peroxidase was quenched for 25 minutes in 0.3% hydrogen peroxidase in methanol. Positive controls and procedures of epitope retrieval and antibody incubation are summarized in Supplementary Table S2. Incubation of FFPE control tissue and cells without primary antibody served as negative controls. Most stainings were performed manually, however, stainings for EGFR and Ki-67 were performed using the BondMax Immunostainer (Menarini Diagnostics, Firenze, Italy) and Bond TM Epitope Retrieval Reagent 2 (Novocastra Laboratories, Newcastle, UK) for antigen retrieval, stainings for CEA, MLH1 and MSH6 using the Autostainer Link (Dako, Glostrup, Denmark) and Target Retrieval Solution (Dako, Glostrup, Denmark) for antigen retrieval, and staining for HIF1α using the catalyzed signal amplification (CSA) system (Dako, Glostrup, Denmark). These stainings were performed according to manufacturer's instructions. Secondary antibodies were visualized using diaminobenzidine (DAB) substrate chromogen system. Slides were counterstained with Mayer's haematoxylin.

### Evaluation of protein expression

Stained TMAs were digitally captured using the Mirax slide scanner system, equipped with a 20x objective with a numerical aperture of 0.75 (Carl Zeiss B.V., Sliedrecht, The Netherlands) and a Sony DFW-X710 Fire Wire 1/3″ type progressive SCAN IT CCD (pixel size 4.65 × 4.65 μm), resulting in an actual scan resolution (effective pixel size in the sample plane) at 20x of 0.23 μm. Stained neoplastic cells or stroma were scored for intensity (categories negative, weak, moderate, strong) and frequency (categories 0–25%, 26–50%, 51–75%, 76–100%) on computer monitors that were calibrated using the Spyder2PRO software (v1.0–16, Pantone Colorvision, Regensdorf, Switzerland). Dedicated TMA scoring software (v1.14.25.1, 3DHISTECH Ltd., Budapest, Hungary) was used for scoring. Core biopsies were independently evaluated by a second investigator, who at time of assessment was unaware of clinicopathological information. For facilitating scoring, a chart with visual analogue scales of staining patterns was used.

### Statistical analysis

Retrospective assessment of the prognostic value was based on methods described previously [32]. Briefly, in a 500-fold cross-validation procedure an optimal cut-off for classifying expression of individual proteins as ‘low’ or ‘high’ was calculated in each training set, based on receiver operating characteristic (ROC) curve analysis for survival data [43–45]. Crude hazard rate ratios (HRRs) for OS were calculated in corresponding validation sets using univariate Cox regression analysis and multivariate Cox regression analysis, performed by stepwise backward regression with *p* > .1 as exclusion criterion and including the following established clinicopathological prognostic variables: primary tumor-to-CRCLM interval < 12 months, lymph node positivity at time of diagnosis of the primary tumor, maximal CRCLM diameter > 5.0 cm, number of CRCLM > 1 and serum CEA level > 200 ng/ml [3]. For each protein, a cross-validated multivariate HRRav was calculated by averaging the multivariate HRRs of the validation sets. The proportion of HRRs in the validation sets that demonstrated a reversed effect compared to the cross-validated HRRav was calculated (i.e. *P* (HRR > 1) or *P* (HRR < 1)). Per candidate biomarker, either the intensity or the frequency immunohistochemistry score with maximum deviation from HRR_av_ = 1 was selected for further analysis (Supplementary Table S3). Classification and regression tree (CART) analysis was used to select the optimal prognostic subset of biomarkers from the total set of biomarkers [46]. Essentially, this is the subset of biomarkers that gives the lowest misclassification error rate for three-year survival in a 10-fold cross-validation procedure. Excluded from survival analyses were patients with unknown or less than two months survival, patients of whom no survival data was available at three years after CRCLM resection or when tissue cores were non-evaluable for technical reasons. OS was visualized using Kaplan-Meier curves and an uncorrected *p*-value was calculated using logrank testing. Patients with missing scores for biomarkers of the final CART tree were also excluded from these analyses. Subsequently, Cox regression analysis was used to obtain a HRR for each CART class compared to the class with the highest survival. CART classes were grouped based on similarity of HRRs [40]. To calculate the *p*-value for the association between class and survival in absence of an external validation dataset, we used a correction procedure based on permutation analysis of the original dataset. In brief, *n* = 10000 permutation datasets were created and for each permutated dataset the optimal classifier and corresponding *p*-value were determined using the procedure as described above. The corrected *p*-value of the CART classifier is the percentage of *p*-values from the permutated analyses that are smaller than the original uncorrected *p*-value. Analysis of variance (ANOVA) was performed to compare the prognostic value of the CART classifier with a model based on the clinicopathological variables and a *p*-value was calculated using chisquare testing. Correlation coefficients between protein expression were calculated using Pearson's correlation test. OS was defined as the time in months after CRCLM resection until death in a follow-up period of up to 10 years. *P* values were considered significant when < .05. Statistical analyses were performed using IBM SPSS Statistics 20.0 software (SPSS Inc., Illinois, USA) and R Statistics 14.0 software (RStudio Inc., Boston, USA). All data reported was REMARK compliant [47].

## SUPPLEMENTARY MATERIALS TABLES AND FIGURES


