# Supplementary material for: A prognostic classifier for patients with colorectal cancer liver metastasis, based on AURKA, PTGS2 and MMP9
Source: Oncotarget. 2015 Oct 20;7(2):2123–34. doi: 10.18632/oncotarget.6188 (PMC4811521; doi:10.18632/oncotarget.6188)
Supplement: Supplementary file 1 [file oncotarget-07-2123-s001.pdf]

A prognostic classifier for patients with colorectal cancer liver metastasis, based on AURKA, PTGS2 and MMP9

Supplementary Materials

Supplementary Table 1: Patient characteristics[1]

	Total Population			Total Population	
**Clinicopathological variable**	***n* /mean /median**	**% /st.dev. /range**	**Clinicopathological variable**	***n* /mean /median**	**% /st.dev. /range**
**General characteristics**			**Liver metastasis characteristics**		
All	507		Age at liver resection		
Gender			mean (st.dev.)	63.3	0.5
male (%)	320	63.1	median (range)	64.3	27.6–83.9
female (%)	187	36.9	Synchronous liver metastasis[Δ]		
OS after liver resection in months			yes (%)[Δ]	221	43.6
mean (st.dev.)	32.2	1.7	no (%)[Δ]	261	51.5
median (range)	27	3.0–212.0	unknown (%)[Δ]	25	4.9
**Primary CRC characteristics**			Distribution liver metastases		
Age at primary CRC resection			left half	90	17.8
mean (st.dev.)	61.9	0.5	segment 1 (%)	18	3.6
median (range)	62.8	24.4–83.3	segment 2 (%)	116	22.9
Primary tumor location			segment 3 (%)	135	26.6
left colon (%)	245	48.3	segment 4 (%)	134	26.4
right colon (%)	95	18.7	right half	259	51.0
rectum (%)	151	29.8	segment 5 (%)	171	33.7
unknown (%)	16	3.2	segment 6 (%)	212	41.8
Histological grade			segment 7 (%)	211	41.6
poorly differentiated (%)	38	7.5	segment 8 (%)	182	35.9
moderately differentiated (%)	288	56.8	both left and right half	146	28.8
well-differentiated (%)	14	2.8	unknown	12	2.4
unknown (%)	167	32.9	Maximal CRCLM diameter in cm[Δ]		
Maximal CRC diameter in cm			mean (st.dev.)	4.1	0.1
mean (st.dev.)	4.4	0.1	median (range)	3.5	0.2–22.0
median (range)	4	0.2–12.0	maximal diameter ≤ 5.0 cm (%)[Δ]	372	73.4
Positive lymph nodes detected[Δ]			maximal diameter > 5.0 cm (%)[Δ]	126	24.8
yes (%)[Δ]	190	37.5	maximal diameter unknown (%)[Δ]	9	1.8
no (%)[Δ]	154	30.4	Number of CRCLM[Δ]		
unknown (%)[Δ]	163	32.1	mean (st.dev.)	2	0.1
Systemic treatment of primary CRC			median (range)	2	1–12
colon cancer			nr of CRCLM = 1 (%)[Δ]	248	48.9
preoperative (%)	9	2.6	nr of CRCLM > 1 (%)[Δ]	253	49.9
perioperative (%)	0	0	nr of CRCLM unknown (%)[Δ]	6	1.2

postoperative (%)	91	26.8	Serum CEA level[Δ]		
none (%)	226	66.5	mean (st.dev.)	101.6	38.5
unknown (%)	14	4.1	median (range)	15	0.5–6625.0
rectal cancer			serum CEA level ≤ 200 ng/ml (%)[Δ]	166	32.7
preoperative (%)	25	16.5	serum CEA level > 200 ng/ml (%)[Δ]	13	2.6
perioperative (%)	1	0.7	serum CEA level unknown (%)[Δ]	328	64.7
postoperative (%)	24	15.9	Extrahepatic metastases		
none (%)	98	64.9	yes (%)	36	7.1
unknown (%)	3	2	no (%)	423	83.4
primary tumor location unknown			unknown (%)	41	8.1
preoperative (%)	1	6.3	Resection in combination with RFA		
perioperative (%)	0	0.0	yes (%)	109	21.5
postoperative (%)	1	6.3	no (%)	357	70.4
none (%)	12	75.0	unknown (%)	41	8.1
unknown (%)	2	12.5	Systemic treatment of liver metastases		
Radiotherapeutic treatment of primary CRC			preoperative (%)	60	11.8
yes (%)	113	22.3	perioperative (%)	12	2.4
no (%)	392	77.3	postoperative (%)	97	19.1
unknown (%)	2	0.4	none (%)	321	63.3
			unknown (%)	17	3.4

OS: overall survival; CEA: carcino-embryonic antigen; RFA: radiofrequency ablation; [Δ]Clinical risk score variable[2]

Supplementary Table 2: Immunohistochemical staining protocols

Protein	Host	Clonality	Incubation	Provider	Epitope retrieval	Detection	Control*
EGFR	mouse	mAb	1:25, 15 min, RT	Novocastra Laboratories (Newcastle, UK)	Bond TM Epitope Retrieval Reagent 2 microwave	NDS	A431 cells
PI3K	rabbit	pAb	1:200, O/N, 4°C	Atlas Antibodies (Stockholm, Sweden)	10 mM citric acid (pH 6) microwave	EV	Caco2 cells
AURKA	mouse	mAb	1:50, O/N, 4°C	Novocastra Laboratories (Newcastle, UK)	10 mM citric acid (pH 6) autoclave	EV	Caco2 cells
Ki-67	mouse	mAb	1:200, 60 min, RT	Dako (Glostrup, Denmark)	Bond TM Epitope Retrieval Reagent 2 microwave	NDS	tonsil
TK1	mouse	mAb	1:100, 60 min, RT	Abnova (Taipei City, Taiwan)	10 mM citric acid (pH 6) microwave	PV	Caco2 cells
KCNQ1	rabbit	pAb	1:200, O/N, 4°C	Santa Cruz Biotechnology (Dallas, USA)	10 mM citric acid (pH 6) microwave	EV	colon
IGF2	rabbit	pAb	1:500, O/N, 4°C	Atlas Antibodies (Stockholm, Sweden)	10 mM citric acid (pH 6) microwave	EV	Caco2 cells
VEGFA	mouse	mAb	1:50, O/N, 4°C	DakoCytomation (Glostrup, Denmark)	10 mM citric acid (pH 6) microwave	PV	Caco2 cells
PDGFRβ	rabbit	mAb	1:50, O/N, 4°C	Cell Signaling Technology (Boston, USA)	10 mM citric acid (pH 6) autoclave	PV	kidney
CEA	mouse	mAb	1:200, 60 min, RT	Biogenex Laboratories (Fremont, USA)	Target Retrieval Solution microwave	EV	colon
MMP9	rabbit	pAb	1:500, O/N, 4°C	Thermo Scientific (Middletown, USA)	10 mM citric acid (pH 6) microwave	EV	placenta
CXCR4	rabbit	pAb	1:1000, O/N, 4°C	Novus Biologicals (Littleton, USA)	10 mM citric acid (pH 6) microwave	EV	lung
CXCL12	mouse	mAb	1:400, O/N, 4°C	R&D Systems (Minneapolis, USA)	10 mM citric acid (pH 6) microwave	EV	Caco2 cells
MLH1	mouse	mAb	1:50, 60 min, RT	BD Biosciences (San Diego, USA)	Target Retrieval Solution microwave	EV	colon
MSH6	mouse	mAb	1:100, 60 min, RT	BD Biosciences (San Diego, USA)	Target Retrieval Solution microwave	EV	colon
PTGS2	rabbit	pAb	1:200, 60 min, RT	Atlas Antibodies (Stockholm, Sweden)	10 mM citric acid (pH 6) microwave	EV	Caco2 cells
SLC2A1	rabbit	pAb	1:600, 60 min, RT	Abcam (Cambridge, UK)	10 mM citric acid (pH 6) water bath	EV	Caco2 cells
HIF1α	mouse	mAb	1:500, 30 min, RT	BD Biosciences (San Diego, USA)	Target Retrieval Solution water bath	CSA	renal cell carcinoma

*Control tissues and cells were formalin-fixed paraffin embedded

mAb: monoclonal antibody; pAb: polyclonal antibody; O/N: overnight; RT: room temperature; EV: Envision Plus (Dako, Glostrup, Denmark); PV: Powervision (Dako, Glostrup, Denmark); NDS: Novocastra Detection System (Novocastra Laboratories, Newcastle, UK); CSA: Catalyzed Signal Amplification system (Dako, Glostrup, Denmark)

Supplementary Table 3: Immunohistochemistry scores with maximum deviation from $HRR_{av} = 1$

	Epithelial							Stromal
	Intensity				Frequency			
**Biomarker**	**Membrane**	**Cytoplasm**	**Nucl.Mem**	**Nucleus**	**Membrane**	**Cytoplasm**	**Nucleus**	**Intensity**
EGFR					X			
PI3K						X		
AURKA				X				
Ki-67							X	
TK1							X	
KCNQ1	X							
IGF2				X				
VEGFA		X						
PDGFRβ								X
CEA	X							
MMP9						X		
CXCR4				X				
CXCL12	X							
MLH1							X	
MSH6							X	
PTGS2			X					
SLC2A1	X							
HIF1α							X	

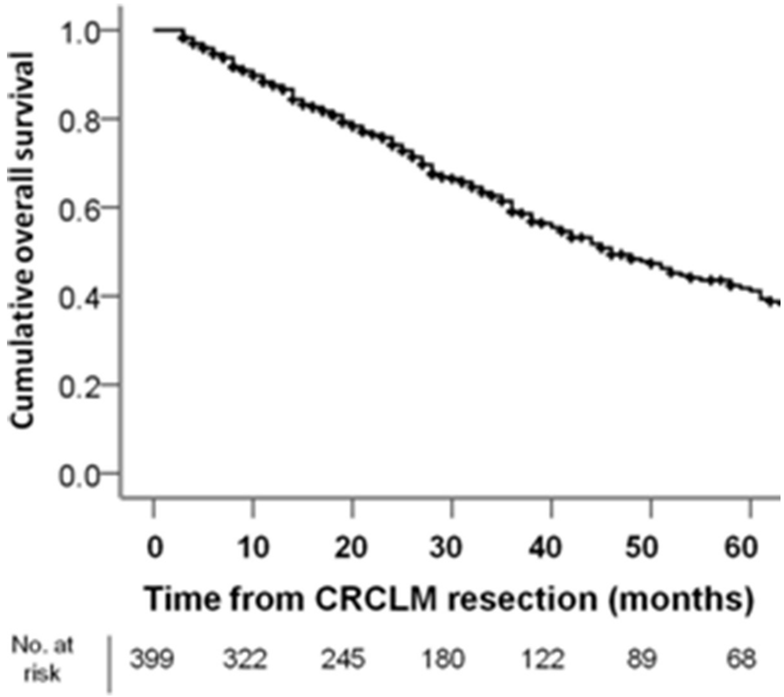

Supplementary Figure 1: Kaplan-Meier graph depicting OS in months of the total study population. Patients with OS less than 2 months and unknown outcome or time of OS were removed from analysis.

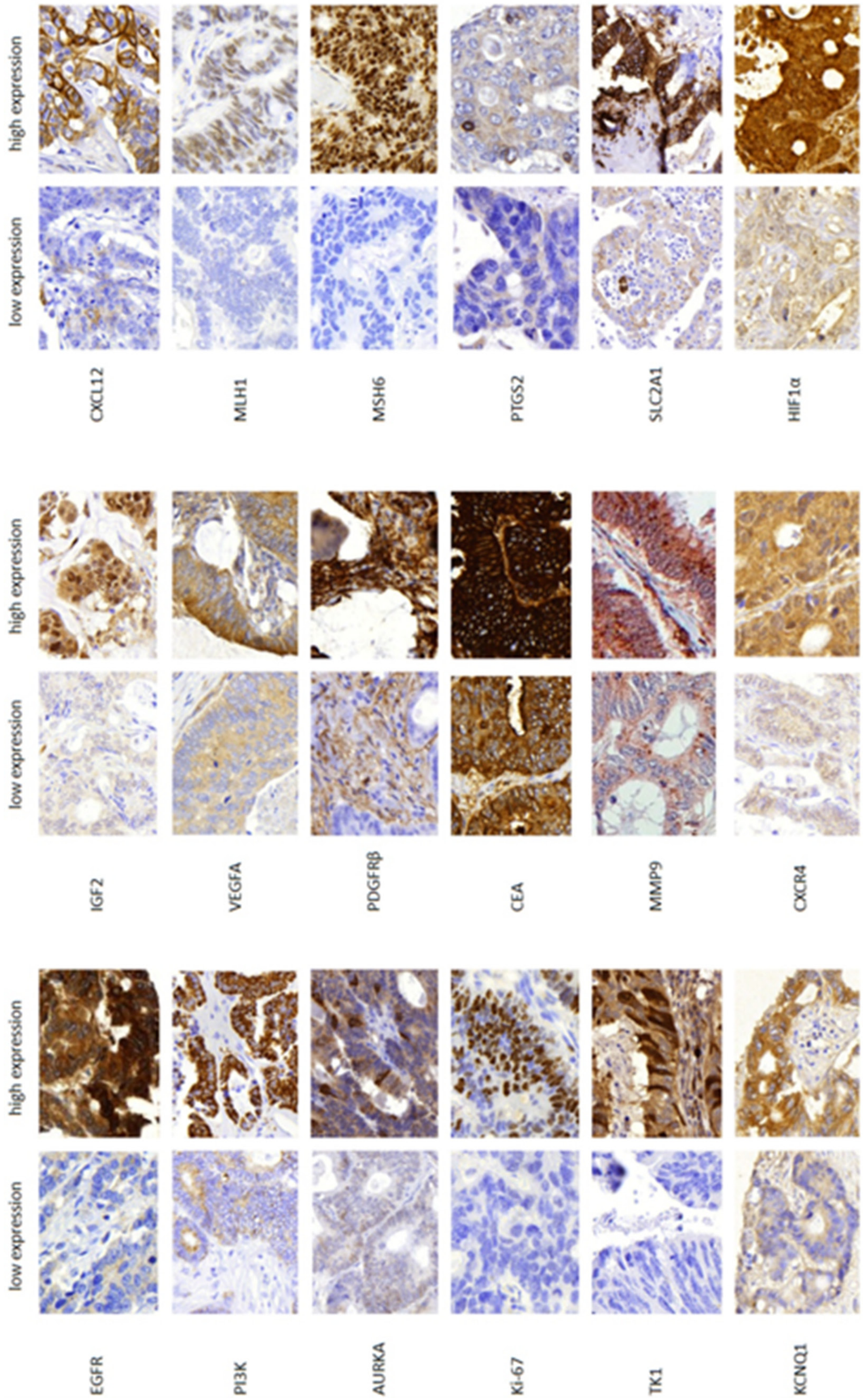

Supplementary Figure 2: Staining examples of candidate biomarker expression in CRCLM.

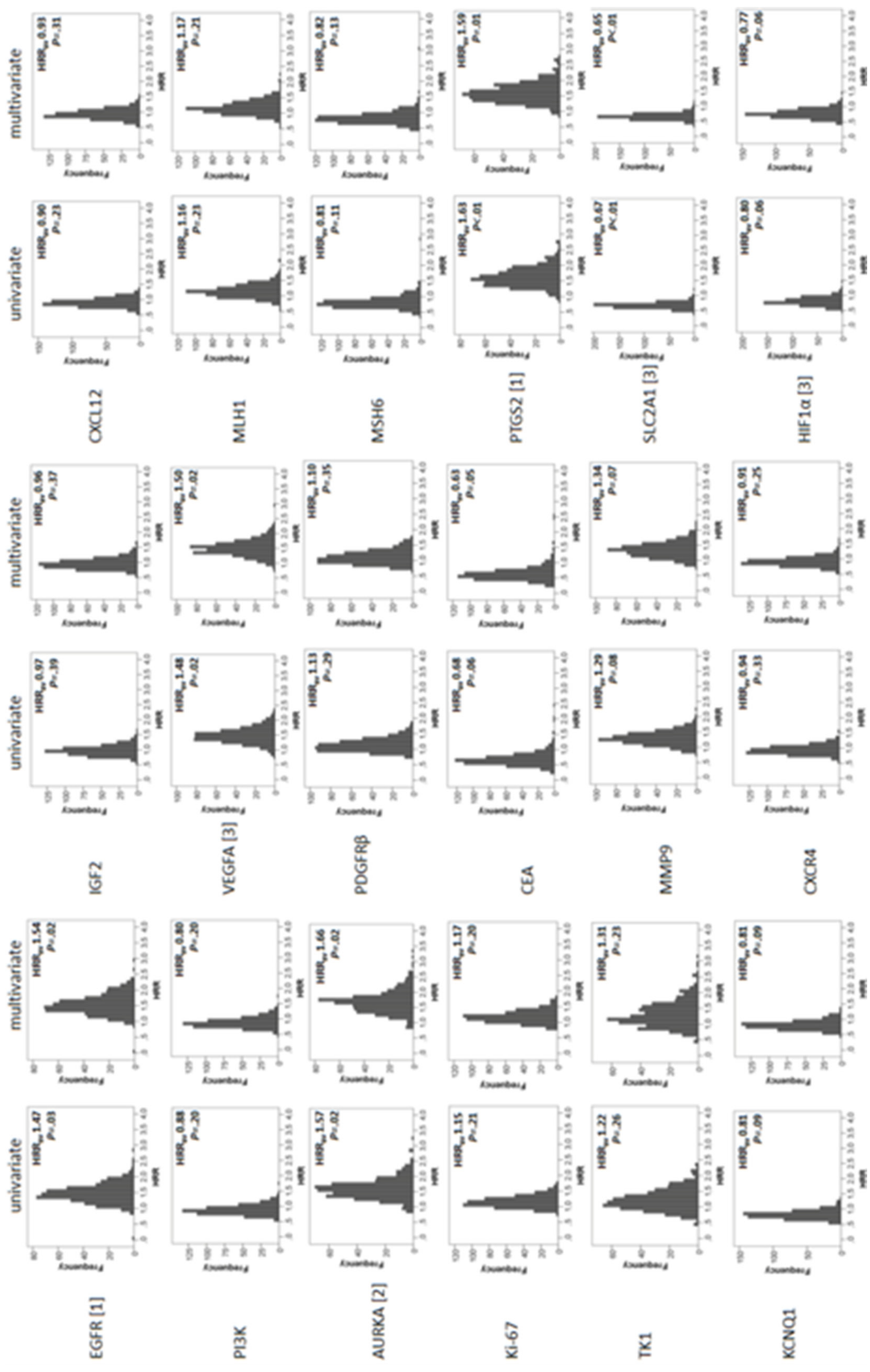

Supplementary Figure 3: Distribution of the cross-validated HRRs after univariate and multivariate analysis of candidate biomarker expression with OS as outcome.

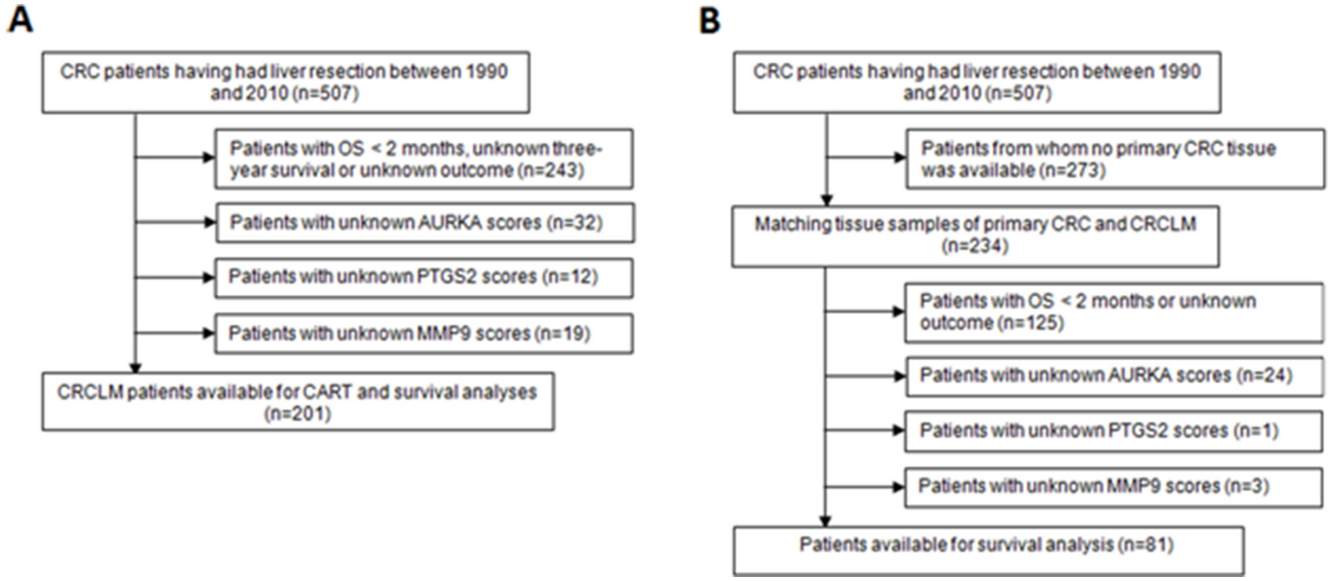

Supplementary Figure 4: Flow diagram of patients in the Classification and Regression Trees (CART) and/or survival analysis using (**A**) patients of whom CRCLM tissue was available and (**B**) patients of whom both CRCLM and primary tumor tissue was available.

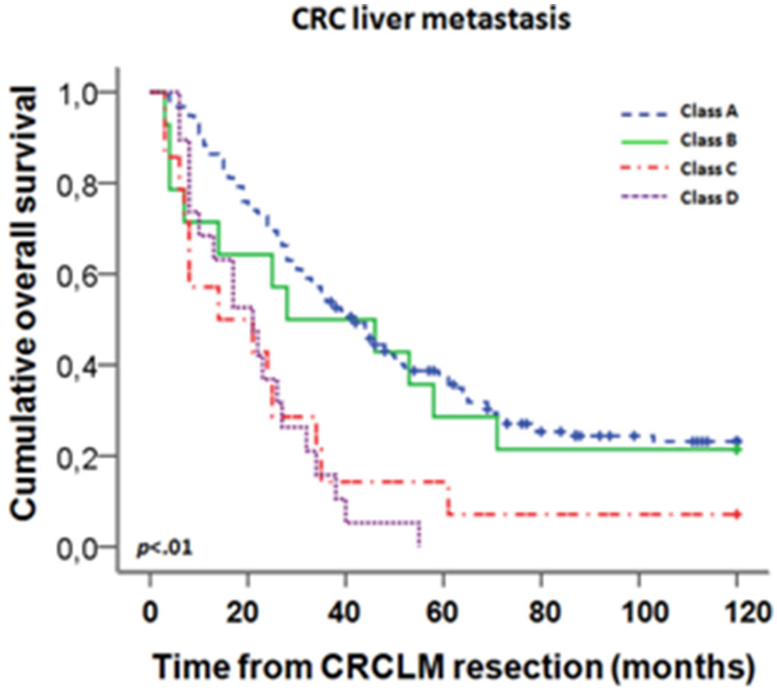

No. at risk							
Class A	154	116	78	49	28	20	15
Class B	14	9	7	4	3	3	3
Class C	14	7	2	2	1	1	1
Class D	19	10	1	0	0	0	0

Supplementary Figure 5: Kaplan-Meier graph depicting OS in months, stratified by the four classes resulting from the CART analysis, based on expression in CRCLM. The *p*-value is the corrected *p*-value as determined by permutation analysis. Excluded from the analysis were patients with unknown or less than two months survival and with unknown expression of AURKA, PTGS2 and MMP9.

REFERENCES

1. Goos JACM, Coupe VMH, Diosdado B, Delis-Van Diemen PM, Karga C, Belien JAM, et al. Aurora kinase A (AURKA) expression in colorectal cancer liver metastasis is associated with poor prognosis. Br J Cancer 2013; 109:2445–52.
2. Fong Y, Fortner J, Sun RL, Brennan MF, Blumgart LH. Clinical score for predicting recurrence after hepatic resection for metastatic colorectal cancer: analysis of 1001 consecutive cases. Ann Surg 1999; 230:309–18.
3. Goos JACM, Hiemstra AC, Coupe VMH, Diosdado B, Kooijman W, is-van Diemen PM, et al. Epidermal growth factor receptor (EGFR) and prostaglandin-endoperoxide synthase 2 (PTGS2) are prognostic biomarkers for patients with resected colorectal cancer liver metastases. Br J Cancer 2014; 111:749–55.
4. Goos JACM, Coupe VMH, Diosdado B, Delis-Van Diemen PM, Karga C, Belien JAM, et al. Aurora kinase A (AURKA) expression in colorectal cancer liver metastasis is associated with poor prognosis. Br J Cancer 2013; 109:2445–52.
5. Goos JACM, Cuba EMV, Coupé VMH, Diosdado B, Delis-Van Diemen PM, Karga C, et al. Glucose transporter 1 (SLC2A1) and vascular endothelial growth factor A (VEGFA) predict survival after resection of colorectal cancer liver metastasis. Ann Surg 2014, In press.
